# Raw nuclear magnetic resonance data of human linker histone H1x, lacking the C-terminal domain (NGH1x), and trajectory data of nanosecond molecular dynamics simulations of GH1x- and NGH1x-chromatosomes

**DOI:** 10.1016/j.dib.2020.105865

**Published:** 2020-06-16

**Authors:** Herna de Wit, Alicia Vallet, Bernhard Brutscher, Leon du Preez, Gerrit Koorsen

**Affiliations:** a University of Johannesburg, South Africa; b Institut de Biologie Structurale Grenoble, France; c University of the Free State, South Africa

**Keywords:** Linker histone, Intrinsically unstructured protein, NMR, BEST-TROSY, Molecular dynamics, Nucleosome, Linker DNA, Core histone

## Abstract

Linker histone H1 plays a vital role in the packaging of DNA. H1 has a tripartite structure: a conserved central globular domain that adopts a winged-helix fold, flanked by highly variable and intrinsically unstructured N- and C-terminal domains. The datasets presented in this article include raw 2D and 3D BEST-TROSY NMR data [1H-15 N HSQC; 15 N and 13C HNCO, HN(CO)CACB, HNCACB, HN(CA)CO] recorded for NGH1x, a truncated version of H1x containing the N-terminal and globular domains, but lacking the C-terminal domain. Experiments were conducted on double-labelled (15 N and 13C) NGH1x in 'low' and 'high salt,' to investigate the secondary structure content of the N-terminal domain of H1x under these conditions. We provide modelled structures of NGH1x (in low and high salt) based on the assigned chemical shifts in PDB format. The high salt structure of NGH1x (globular domain of H1x [GH1x; PDB: 2LSO] with the H1x NTD) was docked to the nucleosome to generate NGH1x- and GH1x-chromatosomes. The GH1x-chromatosome was generated for comparative purposes to elucidate the role of the N-terminal domain. We present raw data trajectories of molecular dynamics simulations of these chromatosomes in this article. The MD dataset provides nanosecond resolution data on the dynamics of GH1x- vs NGH1x-chromatosomes, which is useful to elucidate the DNA binding properties of the N-terminal domain of H1x in chromatin, as well as the dynamic behaviour of linker DNA in these chromatosomes.

Specifications Table

**Table:** 

Subject	Structural Biology
Specific subject area	Chromatin Biology. NMR structural analysis. Molecular dynamics.
Type of data	Figure Table
How data were acquired	NMR data were acquired on Bruker Avance III HD spectrometers, operating at 700 or 950 MHz ^1^H frequency, and equipped with cryogenically cooled triple-resonance (HCN) probes and pulsed z-field gradients at 5 °C (278 K). GH1x (PDB:2LSO) and NGH1x [1] were docked onto a nucleosomal template containing 20 bp of linker DNA and complete core histone tail domains [12] and subjected to energy minimization in YASARA [2,3]. Molecular dynamics simulations were done using GROMACS [4,5] employing the AMBER03 force field [6]. Final energy minimization was done using the steepest descent algorithm in YASARA [7].
Data format	Raw
Parameters for data collection	Experiments were conducted on double labelled (^15^N and ^13^C) NGH1x (residues 1 to 119) in ‘low salt’ [20 mM sodium phosphate buffer (pH 7.0)] or ‘high salt’ [20 mM sodium phosphate buffer + 1 M sodium perchlorate (pH 7.0)]. GH1x and NGH1x were docked to the nucleosome. MDs were performed for 600 ns using the AMBER03 force field. Detailed parameters are provided in the GH1x_and_NGH1x.mdp, GH1x.tpr and NGH1x.tpr files.
Description of data collection	2D ^1^H-^15^N BEST-TROSY spectra [8] were recorded for double-labelled (^15^N and ^13^C) NGH1x (∼0.5 mM) at 5 °C (278 K), 15 °C (288 K) and 25 °C (298 K). A set of 3D BEST-TROSY [9] experiments were recorded and complemented by sequential HADAMAC experiments for amino acid identification [10] followed by the recording of HETNOE spectra. All 3D spectra were recorded at 5 °C (278 K). Molecular dynamics simulations were conducted using GROMACS on the University of the Free State HPC (https://www.ufs.ac.za/ict/adhoc-pages/ict/research-computing).
Data source location	Institution: University of Johannesburg; University of the Free State City/Town/Region: Gauteng; Free State Country: South Africa Institution: Institut de Biologie Structurale (IBS) City/Town/Region: Grenoble Country: France Institution: University of the Free State City/Town/Region: Free State Country: South Africa
Data accessibility	Repository name: Mendeley Data Data identification number: 10.17632/7rjd6r2 76.3Direct URL to data: https://data.mendeley.com/datasets/7rjd6r2 76/3 Repository name: Mendeley Data Data identification number: 10.17632/3kvcfkzpth.3 Direct URL to data: https://data.mendeley.com/datasets/3kvcfkzpth/3
Related research article	de Wit, H., Vallet, A., Brutscher, B. et al. NMR assignments of human linker histone H1x N-terminal domain and globular domain in the presence and absence of perchlorate. Biomol NMR Assign 13, 249-254 (2019). https://doi.org/10.1007/s12104-019-09886-x

Value of the Data

The NMR data provide information on the structure and dynamics of the N-terminal domain (NTD) and globular domain of H1x, whereas the MD trajectory data provide atomic resolution information on the dynamics of these domains in chromatosomes.

Structural biologists, molecular biologists, epigeneticists and computational biologists interested in chromatin structure or intrinsically disordered proteins will find the data useful.
The NMR datasets are useful for the development of NMR pulse sequences for experiments on intrinsically unstructured proteins and/or experiments conducted in high ionic strength conditions. The MD data can serve as a benchmark in the development of molecular dynamics simulations of chromatosomes and for the development of experiments to verify chromatosome models.
Together, the data can be used to evaluate the effect of the N-terminal domain of H1x on the position and orientation of the globular domain of H1x in the nucleosome, chromatosomal protein-DNA interactions, and linker DNA conformation.

## 1 Data description

### 1.1 Raw NMR data

Raw NMR data recorded on Bruker Avance IIIHD spectrometers, operating at 700 or 950 MHz ^1^H frequency, and equipped with cryogenically cooled triple-resonance (HCN) probes and pulsed z-field gradients at 5 °C (278 K) are provided. A description of the data files are provided in the accompanying data repository entry. The raw data provided form the basis of the findings published in [12]. We also offer models of NGH1x (in low and high ionic strength conditions) in PDB format.

### 1.2 Raw MD trajectory data

The data presents nanosecond molecular dynamics simulation trajectories generated in GROMACS. GROMACS molecular dynamics run files are provided in MDP format and the trajectory files in XTC format. Water molecules and ions were not included in the trajectory files. Files containing molecular structures in Gromos87 format (.gro) were also included. Trajectory files span 600 ns, and coordinates and velocities were recorded in 20 ps intervals. Trajectories can be viewed in VMD [11]. We also provide MD quality control data and trajectory energy parameters (Table 1).

**Table 1:** MD data files provided in this article.

Filename	Description
GH1x_and_NGH1x-chromatosome.mdp	MD run file. This file contains the parameters used to run the MD simulations of both GH1x- and NGH1x-chromatosomes.
GH1x-chromatosome.tprNGH1x-chromatosome.tpr	Contain the starting structures, molecular topology, and simulation parameters.
GH1x-chromatosome.groNGH1x-chromatosome.gro	Contain molecular structures in Gromos87 format.
GH1x-chromatosome.xtcNGH1x-chromatosome.xtc	600 ns MD trajectory files, excluding water molecules. Frames were captured at 0.2 ps intervals.

Fig. 1 and Table 2.

**Fig. 1:**
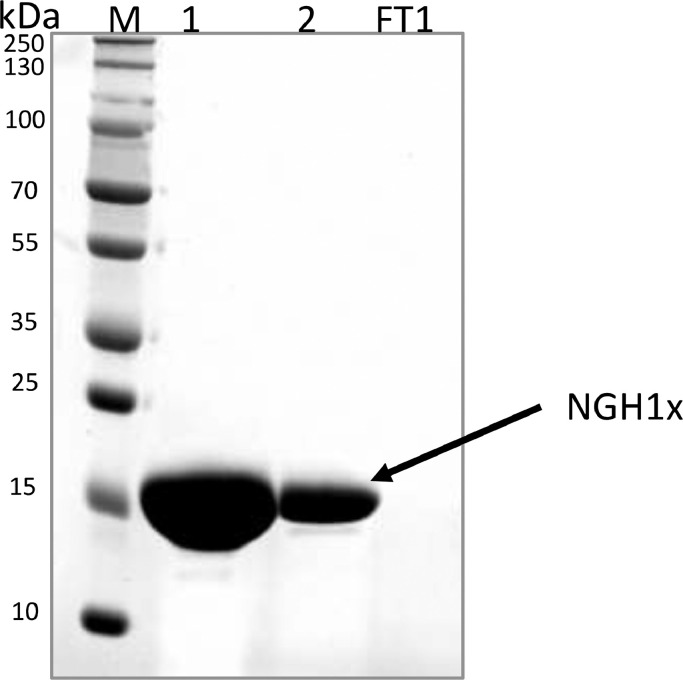
SDS/18% PAGE analysis of purified N^15^/C^13^-NGH1x.

**Table 2:** MD parameters employed (as provided in GH1x_and_NGH1x-chromatosome.mdp file).

Integrator = md	leap-frog integrator
Nsteps = 300,000,000	2 * 300 000 000 = 600 000 ps, 600 ns
Dt = 0.002	2 fs
Output control	
Nstxout = 10,000	save coordinates every 20 ps
Nstvout = 10,000	save velocities every 20 ps
Nstxtcout = 10,000	xtc compressed trajectory output every 20 ps
Nstenergy = 10,000	save energies every 20 ps
Nstlog = 10,000	update log file every 20 ps
Bond parameters	
Continuation = yes	Restarting after NPT
constraint_algorithm = lincs	holonomic constraints
Constraints = *h*-bonds	h-bonds constrained
lincs_iter = 1	accuracy of LINCS
lincs_order = 4	also related to accuracy
Neighbour-searching	
cutoff-scheme = Verlet	
ns_type = grid	search neighbouring grid cells
Nstlist = 40	10 fs
Rlist = 0.8	short-range neighborlist cutoff (in nm)
Rcoulomb = 0.8	short-range electrostatic cutoff (in nm)
Rvdw = 0.8	short-range van der Waals cutoff (in nm)
Electrostatics	
Coulombtype = PME	Particle Mesh Ewald for long-range electrostatics
pme_order = 4	cubic interpolation
Fourierspacing = 0.16	grid spacing for FFT
optimize-fft = yes	
Temperature coupling is on	
Tcoupl = *V*-rescale	modified Berendsen thermostat
tc-grps = Protein Non-Protein	two coupling groups - more accurate
tau_*t* = 0.1 0.1	time constant, in ps
ref_*t* = 300 300	reference temperature, one for each group, in K
Pressure coupling is on	
Pcoupl = Parrinello-Rahman	Pressure coupling on in NPT
Pcoupltype = isotropic	uniform scaling of box vectors
tau_*p* = 2.0	time constant, in ps
ref_*p* = 1.0	reference pressure, in bar
Compressibility = 4.5e–5	isothermal compressibility of water, bar^−1
refcoord_scaling = com	
Compressibility = 4.5e–5	isothermal compressibility of water, bar^−1
refcoord_scaling = com	
Periodic boundary conditions	
Pbc = xyz	3-D PBC
Dispersion correction	
DispCorr = EnerPres	account for cut-off vdW scheme
Velocity generation	
gen_vel = no	Velocity generation is off

**Fig. 2:**
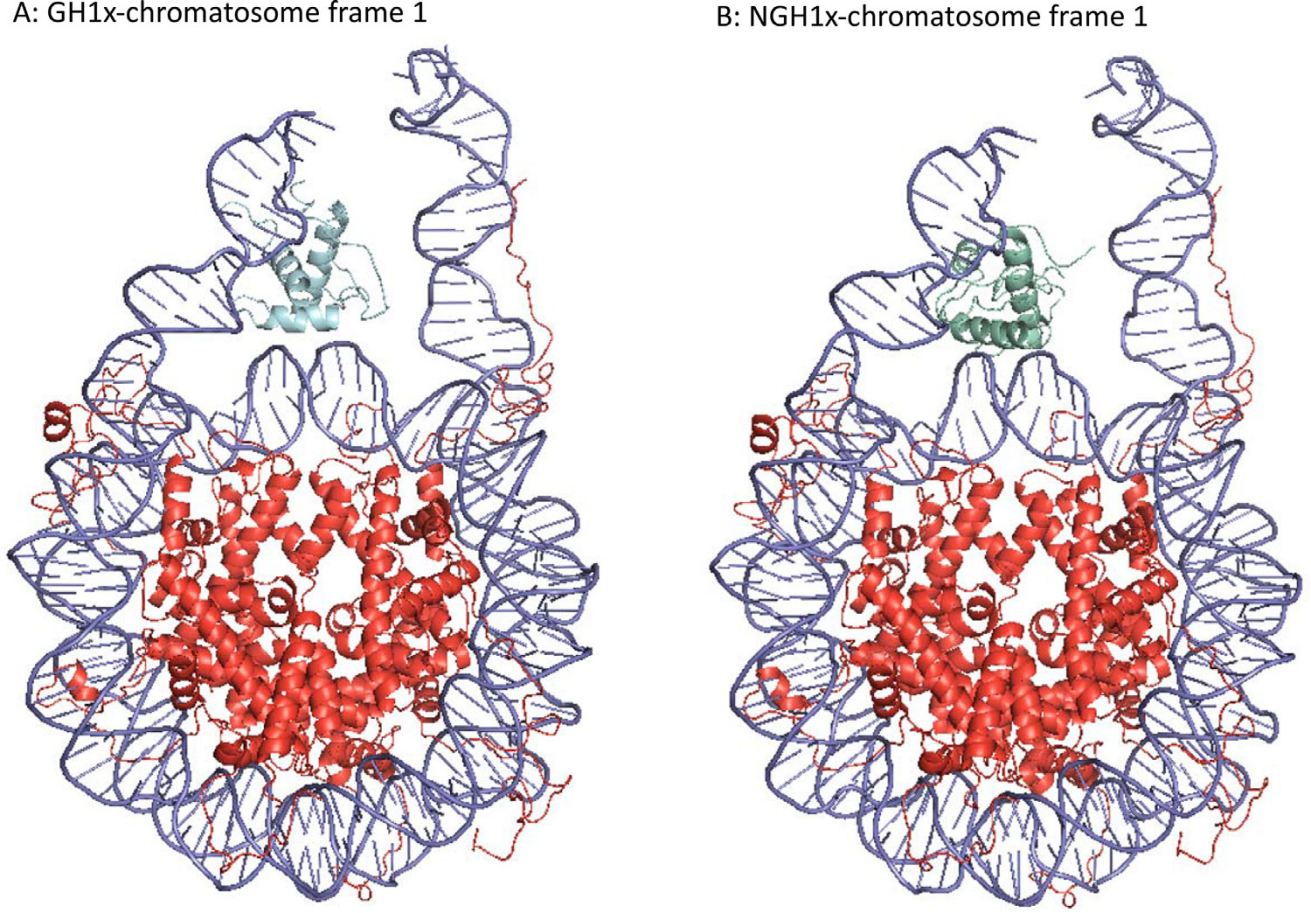
Starting GH1x- and NGH1x-chromatosome structures for MD simulations.

The MD simulations data presented in this manuscript differ from those previously published [1]. The simulations in [1] formed part of the docking procedure to generate the starting structures (Fig. 2) for the extensive MD simulations reported in this article.

Lane M represents a protein marker (Precision Plus Protein™ Unstained Protein Standards, BioRad). Purified protein samples were loaded in lane 1 (1 μL, undiluted sample) and lane 2 (1 μL, 13 x diluted sample). Flow-through from the spin concentrator was loaded in the lane labelled 'FT1′. The gel was stained by Coomassie Brilliant Blue R-250. NGH1x migrated slower than expected due to its highly basic nature.

GH1x-chromatosome frame 1 (A) and NGH1x-chromatosome frame 1 (B) are illustrated. DNA is shown in light purple, core histones, and core histone terminal tails in red, GH1x in light blue, and NGH1x in green cyan. Cartoon structures were rendered with PyMOL.

## 2 Experimental design, materials, and methods

### 2.2 NMR spectroscopy

The generation, expression, and purification of NGH1x [human H1x residues 1- 120] were performed as reported in [12]**.** Briefly, a pET22b(+)-based expression construct was employed, into which a stop codon was inserted 5′ to the vector's 6x His-tag to achieve expression of the untagged protein. Expression of NGH1x was achieved in *E. coli* BL21DE2 cells, and NGH1x was purified using a three-step FPLC procedure that incorporated hydrophobic interaction chromatography (HIC), ammonium sulphate precipitation and ion-exchange chromatography (IEX) [13].

NMR experiments were performed as described [12]. Two-dimensional (2D) 1H–15 N BEST-TROSY spectra, as well as three-dimensional (3D) HNCO, HN(CO)CACB, HNCACB, HN(CA)CO and HADAMAC, were recorded on Bruker Avance IIIHD spectrometers (operating at 700 or 950 MHz ^1^H frequency).

Chemical shift assignments of ^1^H, ^13^C, and ^15^N has been deposited into BMRB (http://www.bmrb.wisc.edu/) with accession number 27,699 for NGH1x in high ionic strength (http://www.bmrb.wisc.edu/data_library/summary/index.php?bmrbId=27699), and 27,700 for NGH1x in low ionic strength (http://www.bmrb.wisc.edu/data_library/summary/index.php?bmrbId=27700).

Assigned spectra of NGH1x in low- and high ionic strength conditions based on the raw data provided here have been published [12].

The structure of NGH1x under low- and high salt conditions was modelled using TALOS+ and CS-ROSETTA.

### 2.3 Molecular dynamics simulations

GH1x (PDB: 2LSO) and the high salt structure of NGH1x was docked to a nucleosomal template containing complete core histone tails and 20 bp of linker DNA [19] using the docking algorithm described in [1]. The GH1x-chromatosome was generated for comparative purposes. The resulting GH1x- and NGH1x-chromatosomes served as starting structures for the MD simulations reported here. Energy minimizations (EM) of starting structures were done in YASARA using the AMBER03 force field and long-range electrostatics. Each starting structure was placed within a rectangular simulation cell (187.30 Å x 187.30 Å x 187.30 Å), and the system was solvated with 203 464 explicit TIP3P solvent molecules.

MD simulations were performed at the University of the Free State (UFS) High-Performance Computing (HPC) Cluster using GROMACS v 4.6.7. The AMBER03 all-atom force field and TIP3P water model [14] were used. Periodic boundary conditions were applied, and long-range electrostatics were treated with the PME method (grid spacing: 0.16 nm and 0.8 nm cut-off). P-LINCS [15] was implemented to constrain bonded hydrogen motions, and the SETTLE algorithm [16] was used to limit solvent motions.

An initial EM run was performed to remove steric hindrance and irregular bond lengths in the starting structure. Two simulation runs were performed with nucleosome positions constrained for further equilibration: a 2 ns NVT simulation to equilibrate the temperature of the system to 300 K using the velocity rescaling thermostat [17], and a 20 ns NPT simulation to equilibrate the pressure of the system to 1 bar using the Parrinello-Rahman barostat [18].

GH1x- and NGH1x-chromatosome production runs were individually performed over 600 ns with a time step for integration of 2 fs at 300 K and 1 bar on the unconstrained nucleosome. Trajectory frames were saved every 0.2 ps to produce a total of 30 000 simulation frames. The production runs were each performed on a total of 192 cores and ran for 86 days on the HPC Cluster at the UFS. EM simulations were conducted with the steepest descent algorithm using GROMACS. Preliminary MD quality control analysis was performed in GROMACS. RMSD and drifts were calculated with full precision.

Following MD simulations, energy minimizations were again performed using a steepest descent algorithm in GROMACS. The procedure terminated after energy conversion.

We provide MD quality control data and energy parameters in the associated Mendeley data entry.

## Declaration of Competing Interest

The authors declare that they have no known competing financial interests or personal relationships which have, or could be perceived to have, influenced the work reported in this article.

## Appendix Supplementary materials

Image, application 1
